# sInterBase: a comprehensive database of *Escherichia coli* sRNA–mRNA interactions

**DOI:** 10.1093/bioinformatics/btad172

**Published:** 2023-04-12

**Authors:** Shani Cohen, Eden Maximof, Shay Rokach, Mor Tadeski, Isana Veksler-Lublinsky

**Affiliations:** Department of Software and Information Systems Engineering, Ben-Gurion University of the Negev, David Ben-Gurion Blvd. 1, Beer-Sheva 8410501, Israel; Department of Software and Information Systems Engineering, Ben-Gurion University of the Negev, David Ben-Gurion Blvd. 1, Beer-Sheva 8410501, Israel; Department of Software and Information Systems Engineering, Ben-Gurion University of the Negev, David Ben-Gurion Blvd. 1, Beer-Sheva 8410501, Israel; Department of Software and Information Systems Engineering, Ben-Gurion University of the Negev, David Ben-Gurion Blvd. 1, Beer-Sheva 8410501, Israel; Department of Software and Information Systems Engineering, Ben-Gurion University of the Negev, David Ben-Gurion Blvd. 1, Beer-Sheva 8410501, Israel

## Abstract

**Summary:**

sInterBase is a comprehensive and easy-to-operate web-based platform for mining experimentally identified sRNA–mRNA interactions in *Escherichia coli*. Interactions in the database are annotated with an interaction duplex and a set of descriptive features. sInterBase provides advanced functionality, such as flexible search based on various criteria, statistical analysis via charts, browsing, and downloading interactions for further use.

**Availability and implementation:**

sInterBase is available at https://sinterbase.cs.bgu.ac.il/.

## 1 Introduction

Bacterial small RNAs (sRNAs) are relatively short noncoding RNA molecules (∼50–500 nt) that play a significant role in the post-transcriptional regulation of various bacterial functions (Bloch et al. 2017). The sRNAs act as regulators either by binding specific proteins to alter their activity or by base-paring with target mRNAs through partially complementary interactions (Storz et al. 2011; Wagner and Romby 2015). In many bacterial species, these interactions are mediated by proteins, e.g., Hfq (Gottesman and Storz 2011), RNase E (Park et al. 2021), ProQ (Melamed et al. 2020), and CsrA (Hör et al. 2020).

The discovery of bacterial sRNA regulation mechanism is much dependent on uncovering their true mRNA targets, and relies on experimental interactions data along with computational methods such as *IntaRNA* (Busch et al. 2008), *CopraRNA* (Wright et al. 2013), and *TargetRNA2* (Kery et al. 2014). To date, several databases provide experimentally identified sRNA–mRNA interactions. The sRNATarBase 3.0 contains mainly *Escherichia coli(E. coli)* interactions, collected manually from the literature (Wang et al. 2016); however, its latest release was in 2015. The RILseqDB contains Hfq-mediated interactions from two high-throughput experiments determined by the RIL-seq methodology (Melamed et al. 2016). Interactions recovered by recent high-throughput experiments such as (Iosub et al. 2020; Melamed et al. 2020) as well as benchmarking datasets such as (Wright et al. 2013; Pain et al. 2015; Gelhausen et al. 2019) are currently not available in any database; instead, they can be downloaded in excel or pdf format from each publication. Beyond the decentralization of interactions’ data reported in various experiments and sources, data representations are inconsistent in genome versions, sRNA and mRNA names, etc., and frequently lack the RNA sequences and/or the interaction duplex.

To enable the effective use of sRNA–mRNA interactions data we developed **sInterBase**, a comprehensive web-based platform for mining experimentally identified sRNA–mRNA interactions in bacteria. It currently compiles data from all available sources for *E.coli* strain K-12 MG1655 and includes 9990 interactions of unique 61 sRNAs and 2095 mRNAs. Each interaction is annotated with an interaction duplex and a set of descriptive features. **sInterBase** provides advanced functionality, such as flexible search based on various criteria, statistical analysis via charts, browsing, and downloading interactions for further use.

## 2 Implementation

**sInterBase** was developed as a single-page application on a Linux server using Python’s FastAPI at the back end, and Javascript’s React at the front end. The PostgreSQL object-relational database system was used for data storage and management. We used the CSS styling language, React Bootstrap, and MUI libraries to create user-friendly interfaces.

### 2.1 Data

The current version includes sRNA–mRNA interactions data of a single, most studied, *E.coli* bacterial strain K-12 MG1655, collected from seven sources listed in Supplementary Table S1. The data are largely divided into interactions (I) and non-interactions (N), i.e., pairs that interact or do not interact, respectively. Non-interaction pairs are designated as such if no sRNA-dependent regulation of the mRNA was observed in the original source. Importantly, regulation was tested under specific lab conditions. For simplicity, we refer to all entries as interactions and use an interaction label (I or N) to distinguish between both types.

#### 2.1.1 Data preprocessing

We downloaded all genes (mRNAs) and noncoding RNAs of *E.coli K12 MG1655* (NC_000913.3) from the EcoCyc database (Keseler et al. 2021), including metadata, and matched each sRNA and mRNA in the interactions’ data to their registered entry in EcoCyc. Next, we handled interactions with inconsistent labels (i.e., sRNA–mRNA pairs that were reported both as interaction and non-interaction in different sources) and converted all the genomic coordinates to the genome version NC_000913.3. Then, we filtered out sRNA fragments whose start and/or end coordinate is located far from the sRNA molecule’s ends. In addition, we filtered out interactions in which the sRNA and/or mRNA fragments were shorter than 15 nucleotides. Altogether, we remained with a final dataset of 9990 interactions (I: 9826, N: 164) of 4332 (I: 4168, N: 164) unique sRNA–mRNA pairs. A detailed description of all steps can be found in the Supplementary Material.

### 2.2 Duplex calculation and feature extraction

We used *RNAup* (Mückstein et al. 2006) to generate an interaction duplex for each entry in the database (see Supplementary Fig. S1). The *RNAup* software takes as input two RNA sequences and identifies the most favorable local interaction site based on minimum free energy (MFE) and accessibility calculations. It was reported to achieve the best performance in terms of true positive rate (TPR) and positive predictive value (PPV) compared to other prediction tools (Umu and Gardner 2016). For each interaction, we calculated various features that are based on energy, duplex structure, and the target’s context. A full description of the duplex generation process and the set of extracted features are elaborated in the Supplementary Information.

### 2.3 User interfaces and functionalities

**sInterBase** provides several functionalities. The browse page displays all database entries in a table. The user can choose to display interactions or non-interactions. Basic information about each interaction and some calculated features are shown on this page. On the search page, the user can select interactions by different categories, e.g., bacterial strain, data source, RNA binding protein (RBP), high-throughput method, sRNA, mRNA, and/or by a variety of calculated features. Choosing multiple categories returns interactions that comply with all criteria. Search can be conducted on interactions or non-interactions. The results are displayed in a table that contains default columns and columns corresponding to the chosen features. These results can be downloaded through the download button with an option to select additional columns extending the available information per interaction. Clicking on the id of an interaction forwards the user to an interaction display that has several sections with detailed information about: (i) interaction, (ii) sRNA, and (iii) mRNA. Pair id unifies all entries of the same sRNA–mRNA pair across all data sources.

The statistics page provides a quantitative summary of the data elements. Most importantly, it offers a statistical analysis of the interactions’ data via various charts. Creating charts involves three steps. First, selecting the type of chart to produce. Next, selecting the features to be displayed in the selected chart. Finally, selecting interactions or non-interactions, with an option to filter them by specific sRNA and/or mRNA. The chart along with the information on the selected chart type, features, and filters is displayed and can be downloaded as a png file. A detailed help page provides information about different options accompanied by screenshots and examples.

## 3 Discussion and conclusions

**sInterBase** is a web-based platform that provides a comprehensive dataset of *E.coli* sRNA–mRNA interactions, with a user-friendly and straightforward interface for mining, visualizing, and examining the interactions and their descriptive features. We believe that this database will facilitate the development of new sRNA target prediction methods, by providing benchmarking data for evaluation and comparison of different methods. We envision some future developments including: extending the interactions data to current and additional *E.coli* strains and other bacterial species, computing additional interaction features, and providing synthetic negative interaction data [to be generated similarly to miRNA field (Ben Or and Veksler-Lublinsky 2021)] to advance machine learning-based methodologies.

## Supplementary Material

btad172_Supplementary_Data

## Data Availability

The data underlying this article are available for download through the sInterBase webserver.
