# Supplementary material for: sInterBase: a comprehensive database of Escherichia coli sRNA–mRNA interactions
Source: Bioinformatics. 2023 Apr 12;39(4):btad172. doi: 10.1093/bioinformatics/btad172 (PMC10121333; doi:10.1093/bioinformatics/btad172)
Supplement: btad172_Supplementary_Data [file btad172_supplementary_data.docx]

**Supplementary Information**

# Data

**sInterBase** contains 9990 sRNA-mRNA interactions from 7 sources as detailed in **Supplementary Table 1**. One should note that the same pair of sRNA-mRNA may appear multiple times in the database in case its interactions were reported in different experimental settings. Such representation keeps the data in its highest granularity, providing complete information about each interaction that can be utilized for different purposes.

**Supplementary Table 1.** Summary of data sources of sRNA-mRNA interactions in *E.coli*

| Serial  no. | Data source  name | High-throughput method | RBP | No. of  sRNA-mRNA interactions | No. of  unique sRNA | No. of  unique mRNA |
| --- | --- | --- | --- | --- | --- | --- |
| 1 | Wright *et al.*, 2013 | - | - | 88 I: 83, N: 5 | 18 | 68 |
| 2 | sRNATarBase3.0 | - | - | 383 I: 224, N: 159 | 43 | 249 |
| 3 | Pain *et al.*, 2015 | - | - | 61 I: 61, N: 0 | 19 | 51 |
| 4 | Melamed *et al.*, 2016 | RIL-seq | Hfq | 2077 I: 2077, N: 0 | 23 | 888 |
| 5 | Gelhausen *et al.*, 2019 | - | - | 101  I: 101, N: 0 | 15 | 86 |
| 6 | Melamed *et al.*, 2020 | RIL-seq | Hfq, ProQ | 5728 I: 5728, N: 0 | 44 | 1519 |
| 7 | Iosub *et al.*, 2020 | CLASH | Hfq | 1552  I: 1552, N: 0 | 40 | 929 |
|  | **Total** |  |  | **9990  I: 9826, N: 164** | **61** | **2095** |

**I** = the number of interaction entries, i.e., sRNA-mRNA pairs reported to interact; **N** = the number of non-interaction entries, i.e., sRNA-mRNA pairs reported to have no interaction. Non-interaction pairs are designated as such if no sRNA-dependent regulation of the mRNA was observed in the original source. Importantly, regulation was tested under specific lab conditions.

## Data Preprocessing

We started with an initial dataset of 12680 sRNA-mRNA interactions and took the following pre-processing steps. First, we downloaded all genes (mRNAs) and non-coding RNAs of *Escherichia coli* *K12 MG1655* (NC_000913.3) from the [EcoCyc](https://ecocyc.org/) database (Keseler et al., 2021), including metadata e.g., accession id, locus tag, common name, etc. **Second**, we matched each sRNA and mRNA in the interactions’ data to its registered entity in EcoCyc, first by accession id or locus tag (if provided), and next by RNA name, considering both the common name and name synonyms provided by EcoCyc. We have succeeded to match RNA information for 11334 out of the initial pool. **Third**, we handled interactions with inconsistent labels (i.e., sRNA-mRNA pairs that were reported both as interaction and non-interaction in different sources), as follows: In case an sRNA-mRNA pair was reported as interaction in one study but was also reported as non-interaction in a benchmarking dataset, we labeled the pair as interaction, as evidence of interaction cancels non-interaction evidence. **Fourth**, we converted all the genomic coordinates of the sRNA and mRNA chimeric fragments (from RIL-seq (Melamed et al., 2016) and CLASH (Iosub et al., 2020)) to the same genome version (NC_000913.3), as follows: (1) Extracting the fragments’ sequences from the source genome version (e.g., NC_000913.2) using the original coordinates provided by the source. (2) Searching each fragment in the complete genome sequence of version NC_000913.3 to find its compatible coordinates. (3) Filtering out interactions that their fragments were mapped to more than a single location in genome version NC_000913.3. Furthermore, in case the source provided both the fragment sequence and fragment‘s coordinates (e.g., CLASH (Iosub et al., 2020)), we have also validated the compatibility between the given fragment sequence and the sequence that is obtained from the genome when using the given fragments’ coordinates and filtered out incompatible interactions. In total 132 interactions were filtered out in this step. **Fifth**, we filtered out sRNA fragments whose start and/or end coordinate is located out of the sRNA molecule with a distance of more than 10 nucleotides from the molecule’s annotated 5’/3’ end. **Sixth**, we filtered out interactions in which the sRNA fragment and/or the mRNA fragment were shorter than 15 nucleotides, remaining with a final dataset of **9990** interactions (I: 9826, N: 164) of 4332 (I: 4168, N: 164) unique sRNA-mRNA pairs.

# Duplex Calculation and Feature Extraction

For each interaction in the database, we defined the sRNA and mRNA input sequences for *RNAup* as follows: In case the source provided the sRNA and mRNA fragments of the interaction, the input sequences were the RNA fragments extended 20 nts upstream and 20 nts downstream, as previously done by Melamed et al. (Melamed et al., 2016). In case the source did not provide the RNA fragments of the interaction, the sRNA input sequence was the entire sRNA molecule, and the mRNA input sequence was defined as -200 nts upstream and +100 nts downstream with respect to the start codon, as previously done by Wright et al (Wright et al., 2013). The *RNAup* software computes an optimal interaction region between the sRNA and mRNA sequences and uses the underlying mechanism of *RNAduplex* to compute an optimal interaction structure (i.e., interaction duplex) for that region. In case *RNAduplex* structure does not coincide with *RNAup* predictions, the software returns an error^[[1]](#footnote-1)^ and no duplex nor features are computed for the interaction. For each interaction that did not return an error by *RNAup*, we have calculated features that are based on energy, duplex structure, and the target’s context. A full description of all calculated features is provided in **Supplementary Table 2**. An example of an interaction duplex and features is presented in **Supplementary Figure 1**. The coordinates shown on the duplex correspond to the positions on the input sequences to RNAup. In case the source provided the sRNA and mRNA fragments of the interaction, the input sequences were the RNA fragments extended 20 nts upstream and 20 nts downstream. In case the source did not provide the RNA fragments of the interaction, the sRNA input sequence was the entire sRNA molecule, and the mRNA input sequence was defined as -200 nts upstream and +100 nts downstream with respect to the start codon.

**Supplementary Table 2.** sInterBase legend

| **Field** | **Description** |
| --- | --- |
| Interaction | |
| Interaction id | sInterBase local id |
| Pair id | sInterBase id for a unique pair of sRNA-mRNA |
| Interaction label | Interaction or non-interaction |
| Data source | Article or database from which the interaction was retrieved |
| Experiment | Experimental setting (table names defined in the original source) |
| High-throughput method | E.g., CLASH, RIL-seq |
| Bacterial strain | Bacterial strain name |
| Chromosome | - |
| RBP | RNA binding protein that mediates the interaction, if known |
| Number of chimeras | Number of chimeras supporting the interaction (data sources e.g., RIL-seq and CLASH) |
| sRNA fragment start | Location on the genome and sequence of the sRNA fragment (relevant to sources e.g., RIL-seq and CLASH) |
| sRNA fragment end |  |
| sRNA fragment strand |  |
| sRNA fragment sequence |  |
| sRNA fragment length |  |
| mRNA fragment start | Location on the genome and sequence of the mRNA fragment (relevant to sources e.g., RIL-seq and CLASH) |
| mRNA fragment end |  |
| mRNA fragment strand |  |
| mRNA fragment sequence |  |
| mRNA fragment length |  |
| sRNA fragment-RNA overlap desc. | Position of the sRNA fragment relative to the sRNA molecule (on the same strand).  Values description:  **Upstream –** Fragment is located upstream of the molecule, with no overlap between them.  **Upstream overlap –** Fragment is located upstream of the molecule and overlaps the molecule from this direction.  **Downstream –** Fragment is located downstream of the molecule, with no overlap between them.  **Downstream overlap –** Fragment is located downstream of the molecule and overlaps the molecule from this direction.  **Fragment within RNA –**The molecule contains the fragment.  **RNA within fragment –** The RNA contains the fragment. |
| sRNA fragment-RNA overlap length | The number of nucleotides that are shared between the sRNA fragment and the sRNA molecule in case of overlap (i.e., upstream overlap, downstream overlap, fragment within RNA, or RNA within fragment). |
| mRNA fragment-RNA overlap desc. | Position of the mRNA fragment relative to the mRNA molecule (values as for sRNA). |
| mRNA fragment-RNA overlap length | The number of nucleotides that are shared between the mRNA fragment and the mRNA molecule in case of overlap. |
| Total energy dg | Energy values computed by *RNAup* |
| Unfolding energy sRNA |  |
| Unfolding energy mRNA |  |
| Hybridization energy |  |
| sRNA interacting area sequence | The sRNA sequence that is involved in the interaction (computed by *RNAup*) |
| sRNA interacting area length | Number of nucleotides in the interacting area of the sRNA, including base pairs, mismatches, and bulges |
| mRNA interacting area sequence | The mRNA sequence that is involved in the interaction (computed by *RNAup*) |
| mRNA interacting area length | Number of nucleotides in the interacting area of the mRNA, including base pairs, mismatches, and bulges |
| Number of base-pairs | Features describing the base-pairing patterns of the *RNAup* duplex formed between the sRNA and the mRNA |
| Number of GC base-pairs |  |
| Number of AU base-pairs |  |
| Number of GU base-pairs |  |
| Number of mismatches |  |
| Number of bulges in the sRNA |  |
| Number of bulges in the mRNA |  |
| Number of max consecutive mismatches |  |
| Number of max consecutive bulges in the sRNA |  |
| Number of max consecutive bulges in the mRNA |  |
| Number of max consecutive  base-pairs - including GU |  |
| Number of max consecutive  base-pairs - excluding GU |  |
| mRNA context - A prop. | The proportion of each NT in the mRNA interacting area extended 20 nts upstream and 20 nts downstream |
| mRNA context - U prop. |  |
| mRNA context - G prop. |  |
| mRNA context - C prop. |  |
| sRNA | |
| sRNA name | The common name of the sRNA |
| sRNA accession id (EcoCyc) | The accession number of the sRNA in the EcoCyc database |
| sRNA locus tag | - |
| sRNA start | Location of the sRNA on the bacterial genome |
| sRNA end |  |
| sRNA strand |  |
| sRNA sequence |  |
| sRNA length |  |
| sRNA alias |  |
| mRNA | |
| mRNA name | The common name of the target gene |
| mRNA accession id (EcoCyc) | The accession number of the gene in the EcoCyc database |
| mRNA locus tag | - |
| mRNA start | Location of the mRNA on the bacterial genome |
| mRNA end |  |
| mRNA strand |  |
| mRNA sequence |  |
| mRNA length |  |
| mRNA alias |  |

**Supplementary Figure 1.** Interaction duplex

Features extracted from the duplex structure below: sRNA interacting area length (=22), mRNA interacting area length (=15), no. of base pairs (=14), no. of GC base pairs (=9), no. of AU base pairs (=4), no. of GU base pairs (=1), no. of mismatches (=1), no. of bulges in the sRNA (=7), no. of bulges in the mRNA (=0), no. of max consecutive mismatches (=1), no. of max consecutive bulges in the sRNA (=7), no. of max consecutive bulges in the mRNA (=0), no. of max consecutive base-pairs - including GU (=8), no. of max consecutive base-pairs - excluding GU (=7).


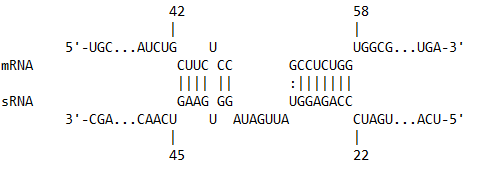


**Figure 1.** An illustration of sRNA-mRNA interaction duplex generated based on *RNAup* outputs for the chimeric input fragments of omrB (sRNA) and accD (mRNA) in *E.coil K12 MG1655*. The coordinates shown on the duplex correspond to the positions on the input sequences to RNAup.

1. <https://www.tbi.univie.ac.at/RNA/RNAup.1.html> [↑](#footnote-ref-1)
